# The Burden of Food Insecurity on Quality of Life in Adults with Diabetes

**DOI:** 10.3390/nu16213602

**Published:** 2024-10-23

**Authors:** Rebekah J. Walker, Joshua K. Egede, Abigail Thorgerson, Elise Mosley-Johnson, Jennifer A. Campbell, Leonard E. Egede

**Affiliations:** 1 Division of Population Health, Department of Medicine, Jacobs School of Medicine and Biomedical Sciences, University at Buffalo, Buffalo, NY 14203, USA; rbwalker@buffalo.edu (R.J.W.);; 2 Center for Advancing Population Science, Medical College of Wisconsin, Milwaukee, WI 53226, USA

**Keywords:** food insecurity, diabetes, quality of life, comorbidities

## Abstract

***Background***: This study aimed to investigate the relationship between food insecurity and physical- and mental-health-related quality of life in adults with diabetes. ***Methods***: Using two years of national Medical Expenditure Panel Survey data (2016–2017), we investigated the relationship between food insecurity and physical-health-related (PCS) and mental-health-related (MCS) quality of life in adults with diabetes. PCS and MCS were measured with the Short-Form 12 health survey and food insecurity was measured with the USDA 10-item adult scale. Analyses were weighted to represent the US adult population. Adjusted linear regression models, including covariates of age, gender, education, race/ethnicity, marital status, region, poverty level, employment status, health insurance, and comorbidities were used. ***Results***: After adjustment, food-insecure adults with diabetes maintained significantly lower quality of life compared to food-secure adults with diabetes (PCS: −3.44, 95%CI −4.63, −2.25; MCS: −5.37, 95%CI −6.68, −4.06). This drop in PCS was larger than the drop for chronic conditions, including arthritis (−3.77, 95%CI −5.02, −2.52), emphysema (−2.82, 95%CI −5.12, −0.53), stroke (−2.63, 95%CI −4.11, −1.15), cancer (−2.59, 95%CI −4.00, −1.17), and heart attack (2.58, 95%CI 4.68, 0.48). Similarly, the drop for MCS was larger than for chronic pain (−2.37, 95%CI −3.24, −1.50) and arthritis (−1.31, 95%CI −2.28, −0.33). ***Conclusions***: Food insecurity was associated with a significant reduction in both physical- and mental-health-related quality of life in adults with diabetes, with a magnitude of effect greater than adjusted estimates for the drop in quality of life for key chronic conditions. Addressing food insecurity through integration of social and medical care may lead to improvements in quality of life for adults with diabetes.

## 1. Introduction

Diabetes is the seventh leading cause of death in the United States, affecting 14.7% of adults [1]. Type 2 diabetes accounts for 90–95% of all diabetes and is associated with significant morbidity due to complications such as cardiovascular and kidney disease, amputations, and nerve damage [1]. Currently, diabetes is associated with excess medical costs of over USD 9500 per person per year, and by 2030, diabetes-related expenses are projected to cost over USD 600 billion annually [1,2]. The social determinants of health, which include social risk factors such as food insecurity, are recognized as a driver of poor health outcomes for adults with diabetes and an area of focus to reduce disparities in clinical outcomes [3].

Beyond clinical and cost implications, diabetes is noted to impact health-related quality of life (HRQOL), a multidimensional metric that encompasses perceived physical and mental health over time [4,5]. Individuals with diabetes often report a lower quality of life due to a multitude of clinical factors such as necessary dietary changes/restrictions, higher burden of annual medical costs, worse glycemic control, diabetes-related complications and comorbidities, and increased depressive symptomology [6,7,8,9,10,11]. In addition, social factors have been noted to influence quality of life in adults with diabetes, including income, education, and social support or social interaction [12,13,14].

Food insecurity, defined as uncertainty of having or inability to acquire enough food for a healthy and active life, is associated with a variety of health outcomes for adults with diabetes [15,16,17,18]. Adults with diagnosed and undiagnosed diabetes are at a greater risk of food insecurity, and food-insecure adults with diabetes have worse glycemic control, poorer diet quality, reduced self-efficacy, increased diabetes distress, and increased medication non-adherence [17,19,20,21,22,23]. Food-insecure adults also have higher healthcare expenditures compared to food-secure adults with chronic conditions, explaining much of this difference [24,25]. While the mechanisms through which food insecurity influences health outcomes are not fully understood, possible pathways include the impact of limited income on accessing health resources, the reliance on less expensive high carbohydrate diets, and stress related to trade-offs due to lower income [3].

The relationship between food insecurity and physical- and mental-health-related quality of life has been noted in other diseases and in the general population [11,26,27,28,29]. For example, food insecurity was found to be associated with worse health-related quality of life and mental health in people living with HIV [30]. In addition, multiple studies noted that individuals with a history of cancer experience higher rates of food insecurity and this is associated with lower general health-related quality of life [31,32,33]. Finally, food insecurity has been associated with poorer health-related quality of life as the number of chronic conditions an individual is diagnosed with increases [34,35,36]. However, how this burden affects quality of life among individuals with diabetes compared to other chronic illnesses remains unknown.

Therefore, the aim of this study was to investigate the relationship between food insecurity and physical- and mental-health-related quality of life in adults with diabetes using a nationally representative dataset, controlling for other comorbidity conditions. We hypothesized that physical- and-mental-health-related quality of life would be significantly lower in food-insecure vs. food-secure adults with diabetes. In addition, we hypothesized that the decrease in quality of life among food-insecure adults with diabetes would be worse than the decrease due to common chronic conditions such as stroke, emphysema, heart disease, and cancer after adjusting for relevant covariates.

## 2. Methods

### 2.1. Sample

The 2016 and 2017 full-year consolidated files and food security files from the household component files of the Medical Expenditure Panel Survey (MEPS) were analyzed. MEPS is a national survey that collects data from U.S. citizens and their families on items such as health services, employment, and insurance. The survey began in 1996 and is overseen by the Agency for Healthcare Research and Quality (AHRQ) [37]. Food insecurity questions were added in 2016 and 2017; however, these were removed after those years of data collection. Therefore, the 2016 and 2017 MEPS dataset was used in this analysis.

As the focus of this analysis was adults with diabetes, the sample used in this study comprised adults 18 and older who were included in the food insecurity file and who reported being diagnosed with diabetes. The unweighted sample comprised 3588 individuals (32,948,078 weighted).

### 2.2. Outcome—Quality of Life

The two primary outcomes were the physical component (PCS) and mental component (MCS) of quality of life. These are continuous measures where higher scores indicate a higher health-related quality of life. MEPS administers the Short-Form-12 Health Survey, Version 2, which assesses four physical domains (functioning, physical role, bodily pain, and general self-rated health) and four mental health domains (vitality, social functioning, emotional role, and mental health). Twelve questions were asked with a 4-week look-back period and then scored by AHRQ using the standard scoring algorithm, which scaled to a population mean of 50 and standard deviation of 10 [37]. The questions participants were asked, scoring algorithm, and accuracy of estimates were reported by MEPS via Methodology Report #15 [38].

### 2.3. Food Insecurity

The primary independent variable was household food insecurity. This variable was scored based on the USDA 10-item Adult Food Security Scale and prior work conducted by Dean et al. to provide comparability of the MEPS questions with the USDA look-back period [24,27]. A raw score was created by adding the affirmative answers to the following prompts:

1. “How often in the last 30 days has anyone in the household worried whether food would run out before getting money to buy more?” Score of 1 if the participant responded ‘often’ or ‘sometimes’.
2. “How often in the last 30 days did the food purchased not last and the person/household didn’t have money to get more?” Score of 1 if the participant responded ‘often’ or ‘sometimes’
3. “How often in the last 30 days could the person/household not afford to eat balanced meals?” Score of 1 if the participant responded ‘often’ or ‘sometimes’.
4. “In the last 30 days, did the person/household reduce or skip meals because there wasn’t enough money for food?” Score of 1 if the participant responded ‘yes’.
5. “How many meals were skipped in the last 30 days?” Score of 1 if the participant responded with 3+ days.
6. “In the last 30 days, did the person/household ever eat less because there wasn’t enough money for food?” Score of 1 if the participant responded ‘yes’.
7. “In the last 30 days, was the person/household ever hungry but didn’t eat because there wasn’t enough money for food?” Score of 1 if the participant responded ‘yes’.
8. “In the last 30 days, did anyone in the household lose weight because there wasn’t enough money for food?” Score of 1 if the participant responded ‘yes’.
9. “In the last 30 days, did anyone in the household not eat for a whole day because there wasn’t enough money for food?” Score of 1 if the participant responded ‘yes’.
10. “How many days in the last 30 days did anyone in the household not eat for a whole day because there wasn’t enough money for food?” Score of 1 if the participant responded with 3+ days.

Since the USDA scale uses the past year, whereas the MEPS questions uses the past 30 days, scores for questions 5 and 10 were adjusted based on the prior work conducted by Dean et al. so that those who answered 3+ days to “how many meals were skipped in the last 30 days?” and “how many days in the last 30 days did anyone in the household not eat for a whole day because there wasn’t enough money for food?” had an additional 1 added to their overall score. The raw score was then categorized into food security (a score of 0–2) and food insecurity (a score of 3–11). If individuals refused to answer, or responded with “I don’t know” or “not ascertained” to all food insecurity questions, they were coded as missing.

### 2.4. Covariates

Based on possible confounders noted in the literature and the interest in understanding the role of food insecurity in comparison to multiple comorbidities, a number of covariates were included in the adjusted analyses. The covariates included categorical age in years (18–44, 45–64, and 65+), gender (male, female), education (less than high school, high school attainment, and college or above), race/ethnicity (Non-Hispanic White, Non-Hispanic Black, Hispanic, other), marital status (married, widowed/divorced/separated, never married), region (Northeast, West, South, Midwest), poverty level (poor/negative income, near-poor income, low income, middle income, or high income), employment status (employed or unemployed), health insurance (private, public, or uninsured), and self-reported comorbidities (stroke, high blood pressure, emphysema, chronic pain, arthritis, asthma, high cholesterol, coronary heart disease, angina, heart attack, heart disease, other heart disease, bronchitis, or cancer). Poverty level was defined by the MEPS dataset using the family income to poverty ratio, considering the family size and age of the head of household. Negative or poor indicates individuals/families with income less than or equal to the poverty line, near-poor indicates a poverty line through 125%, low income indicates 125–200%, middle income indicates 200–400%, and high income indicates over 400% of the poverty line.

### 2.5. Statistical Analysis

All analyses were completed in Stata version 15. A *p*-value less than 0.05 was considered statistically significant. Weighting followed MEPS documentation using the svy function in Stata. Summary characteristics were reported as the percentage for categorical variables and mean with 95% confidence interval (CI) for numeric variables. Chi-square tests were used to compare unadjusted PCS and MCS scores for individuals reporting food security and food insecurity. Unadjusted linear regression models were run separately on the PCS and MCS outcomes with the binary food insecurity as the primary independent variable. Finally, both models were adjusted for age, gender, education, race/ethnicity, marital status, region, poverty level, employment status, health insurance, and comorbidities.

## 3. Results

Table 1 provides sample demographics by food insecurity status. In this sample, 86.4% of adults with diabetes had food security while 13.6% were food insecure. There were significant differences between those with food security and food insecurity by demographics: age, gender, education race/ethnicity, marital status, poverty level, insurance status, and employment (*p*-values < 0.001).

Table 2 presents the means and 95% confidence intervals for the physical component summary (PCS) and mental component summary (MCS) by food insecurity status. The average score for PCS was 42.2 for those with food security and 35.9 for those with food insecurity. The average score for MCS was 51.5 for those with food security and 42.3 for those with food insecurity.

Table 3 displays the unadjusted and adjusted linear regression models for the PCS outcome. The unadjusted model showed those with food insecurity had a 6.31 decrease, on average, in PCS compared to those with food security (β = −6.31, 95% CI = [−4.83, −7.80], *p*-value < 0.001). When controlling for demographics and comorbidities, those with food insecurity had a 3.44 decrease, on average, in PCS compared to those with food security (β = −3.44, 95% CI = [−2.25, −4.63], *p*-value < 0.001). After adjustment, the drop in PCS due to food insecurity remained larger than for common chronic conditions, including stroke (β = −2.63, 95% CI = [−1.15, −4.11], *p*-value < 0.01), high blood pressure (β = −1.51, 95% CI = [−0.30, −2.72], *p*-value < 0.05), emphysema (β = −2.82, 95% CI = [−0.53, −5.12], *p*-value < 0.05), arthritis (β = −3.77, 95% CI = [−2.52, −5.02] *p*-value < 0.001), heart attack (β = 2.58, 95% CI = [0.48, 4.68] *p*-value < 0.05), other heart disease (β = −1.39, 95% CI = −[0.09, −2.70], *p*-value < 0.05), and cancer (β = −2.59, 95% CI = [−1.17, −4.00] *p*-value < 0.001). Only the relationship between chronic pain and PCS was larger relative to food insecurity (β = −4.50, 95%CI [−5.50, −3.50], *p*-value < 0.001).

Table 4 displays the unadjusted and adjusted linear regression models for the MCS outcome. The unadjusted model showed those with food insecurity had an 8.75 decrease, on average, in MCS compared to those with food security (β = −8.75, 95% CI = [−7.45, −10.06], *p*-value < 0.001). When controlling for demographics and comorbidities, those with food insecurity had a 5.37 decrease, on average, in PCS compared to those with food security (β = −5.37, 95% CI = [−4.06, −6.68], *p*-value < 0.001). After adjustment, the drop in MCS remained larger for chronic conditions, including chronic pain (β = −2.37, 95% CI = [−1.50, −3.24], *p*-value < 0.001) and arthritis (β = −1.31, 95% CI = [−0.33, −2.28], *p*-value < 0.01).

## 4. Discussion

Using a nationally representative sample of adults with diabetes, we found food insecurity was associated with a significant reduction in both physical- and mental-health-related quality of life above and beyond having diabetes alone. The magnitude of effect in adjusted models was greater than adjusted estimates for the drop in quality of life for key chronic conditions including stroke, emphysema, heart disease, and cancer. Based on these findings, food insecurity is an underappreciated driver of poor quality of life in adults with diabetes. As such, in addition to improving clinical care for diabetes, this study suggests that addressing food insecurity for adults with diabetes may lead to improvements in quality of life. In addition, based on these findings, studies should incorporate the collection of quality-of-life measures to investigate the impact of interventions on both clinical and patient-reported outcomes over time.

This study adds to the current literature by highlighting the significant burden of food insecurity for adults with diabetes beyond the impact on health behaviors and clinical outcomes noted in prior research. When comparing key chronic conditions, health-related quality of life scores found in this study for adults with diabetes and food insecurity were lower than scores for PCS and MCS found previously in studies on stroke, arthritis, and heart disease, and lower than scores for MCS found in prior studies on patients with emphysema [38,39,40,41]. Using nationally representative data in this study, after adjustment for demographics, insurance, and chronic conditions, we found that PCS for food-insecure individuals with diabetes dropped by 3.4 points, whereas the significant drops in PCS for chronic conditions ranged from 1.4 for other heart disease to 3.7 for arthritis. Only chronic pain (with a significant drop of 4.5 in PCS) was higher than the drop in PCS seen for adults with food insecurity. Similarly, we found that MCS for food-insecure individuals with diabetes dropped by 5.3 points, whereas significant drops for MCS for chronic conditions ranged from 1.3 for arthritis to 2.4 for chronic pain. Therefore, our findings suggest that food insecurity decreased both physical- and mental-health-related quality of life above and beyond what you would expect for chronic conditions. Quality of life scores for comorbidities were similar to or higher than in prior literature [38,39], suggesting the burden noted was not particular to the sample in this study. Our study also found quality of life scores to be lower for adults with diabetes and food insecurity compared to what previous literature has found for individuals facing food insecurity or diabetes alone, suggesting the combination of both food insecurity and diabetes is particularly burdensome [39,42,43].

This study and the existing literature on the importance of quality of life highlight the need for understanding mechanisms that underlie relationships seen between social risk factors, such as food insecurity and quality of life in adults with diabetes. As care models move towards a more holistic approach to health and wellness, multicomponent interventions that incorporate both medical and social factors may be necessary to address the complex intersection of disease management and the role of social factors such as food insecurity on quality of life [44]. Standards of care for diabetes management emphasize the centrality of quality of life for self-care and optimizing metabolic control [45]. As evidence mounts highlighting the compounding burden of social risks such as food insecurity on quality of life and health outcomes for adults with diabetes, a greater understanding of mechanisms through which this relationship can be mitigated is also necessary. To effectively address diabetes from a holistic approach accounting for these dimensions of medical and social needs, interventions designed to address food insecurity in populations with diabetes need to emphasize patient-reported outcomes as well as clinical outcomes by measuring quality of life over time. The results of interventions should present the impact on both clinical and patient-reported outcomes, and more work aimed at understanding the pathways through which these relationships exert their effect is needed.

Though this study used a nationally representative dataset, there are limitations worth noting. First, the data are cross-sectional and cannot speak to causality between food insecurity and quality of life. Second, variables that may help explain some of the relationship between food insecurity and quality of life were not available in the MEPS dataset and so were not captured in this analysis. As a result, future work should collect factors such as diet and distress in addition to demographic factors and comorbidities. Finally, self-reported data are subject to recall bias; however, the SF-12 is a multidimensional scale that has been shown to have sufficient validity and reliability in adults with diabetes [40].

## 5. Conclusions

In conclusion, the burden of food insecurity on both physical- and mental-health-related quality of life is underappreciated in adults with diabetes. Given that the size of this effect in fully adjusted models was larger than that of comorbidities, this study suggests that food insecurity alone has an impact independent of and stronger than that of other chronic conditions. Addressing food insecurity via integration of social and medical care will likely lead to improvements in quality of life for adults with diabetes and should be the focus of future work. In addition, incorporation of quality of life in intervention studies incorporating food insecurity will allow investigation into whether social and medical integration of care can improve quality of life for adults with diabetes.

## Figures and Tables

**Table 1:** Sample demographics weighted to be nationally representative, overall and by food security status (n = 3588, N = 32,948,077.5).

	Total	Food Secure	Food Insecure	*p*-Value
Age				<0.001
18–44	10.1%	9.1%	16.9%	
45–64	44.4%	42.6%	55.8%	
65+	45.5%	48.3%	27.3%	
Gender				<0.001
Male	51.1%	53.5%	35.7%	
Female	48.9%	46.5%	64.3%	
Education				<0.001
Less than High School Diploma	17.0%	15.9%	24.0%	
High School Diploma/GED	33.2%	33.0%	34.3%	
College	49.8%	51.1%	41.6%	
Race/Ethnicity				<0.001
Non-Hispanic White	61.3%	63.1%	50.2%	
Non-Hispanic Black	16.9%	15.8%	23.9%	
Hispanic	14.3%	13.6%	18.8%	
Other	7.5%	7.5%	7.0%	
Marital Status				<0.001
Married	46.0%	48.7%	29.3%	
Widowed/Divorced/Separated	41.0%	39.7%	49.5%	
Never Married	12.9%	11.6%	21.2%	
Region				0.15
Northeast	16.2%	16.4%	15.4%	
West	20.3%	20.9%	16.1%	
South	42.1%	41.2%	48.0%	
Midwest	21.4%	21.5%	20.5%	
Poverty				<0.001
Poor/Near Negative	16.7%	13.7%	35.5%	
Near Poor	6.9%	5.8%	13.8%	
Low Income	16.9%	15.9%	23.4%	
Middle Income	26.9%	27.8%	21.1%	
High Income	32.7%	36.8%	6.2%	
Insurance				<0.001
Private	58.9%	62.9%	32.8%	
Public	37.2%	33.6%	59.9%	
Uninsured	4.0%	3.4%	7.3%	
Employment				<0.001
Employed	39.1%	40.9%	27.0%	
Unemployed	60.9%	59.1%	73.0%	
Stroke	11.4%	11.1%	13.6%	0.16
High Blood Pressure	78.3%	78.6%	76.2%	0.40
Emphysema	5.6%	4.8%	11.2%	<0.001
Chronic Pain	58.8%	57.8%	65.5%	0.01
Arthritis	54.0%	53.4%	58.1%	0.13
Asthma	15.1%	13.2%	27.3%	<0.001
High Cholesterol	75.8%	76.2%	73.4%	0.30
Coronary Heart Disease	15.9%	16.2%	14.0%	0.30
Angina	6.9%	6.8%	7.8%	0.55
Heart Attack	13.3%	12.9%	16.1%	0.10
Heart Disease	22.3%	22.0%	24.2%	0.36
Other Heart Disease	23.3%	22.8%	26.1%	0.19
Bronchitis	4.6%	3.9%	9.2%	<0.001
Cancer	20.3%	21.0%	15.3%	0.01

**Table 2:** Unadjusted mean quality of life scores based on food security status (mean, 95% confidence interval).

	Physical Health Component (PCS)	Mental Health Component (MCS)
Food Secure	42.2 (41.6, 42.8)	51.5 (51.0, 51.9)
Food Insecure	35.9 (34.5, 37.3)	42.3 (41.5, 43.9)

**Table 3:** Unadjusted and adjusted relationship between food insecurity and physical component of quality of life.

	Unadjusted	Adjusted
	Estimate (95% CI)	Estimate (95% CI)
Food Security Status		
Food Secure (ref)	-	-
Food Insecure	−6.31(−4.83, −7.80) ***	−3.44 (−2.25, −4.63) ***
Age		
18–44 (ref)		-
45–64		−1.54 (−3.12, 0.04)
65+		−0.28 (−2.07, 1.51)
Gender		
Male (ref)		-
Female		−0.59 (−1.70, 0.51)
Education		
<High School (ref)		-
High School Diploma/GED		0.55 (−0.80, 1.89)
College		0.54 (−0.75, 1.84)
Race/Ethnicity		
Non-Hispanic White (ref)		-
Non-Hispanic Black		−0.76 (−1.79, 0.27)
Hispanic		0.07 (−1.40, 1.54)
Other		0.01 (−1.67, 1.70)
Marital Status		
Married (ref)		-
Widow/Divorce/Separated		−0.08 (−1.24, 1.08)
Never Married		0.21 (−1.36, 1.78)
Region		
Northeast (ref)		-
West		−1.76 (−0.03, −3.49) *
South		−1.61 (−0.23, −2.99) *
Midwest		−1.79 (−0.31, −3.27) *
Poverty		
Poor/Near Negative (ref)		-
Near Poor		−0.50 (−2.21, 1.20)
Low Income		0.22 (−1.19, 1.62)
Middle Income		1.81 (0.39, 3.24) *
High Income		3.36 (1.74, 4.98) ***
Insurance		
Private (ref)		-
Public		−0.91 (−2.10, 0.29)
Uninsured		1.22 (−0.57, 3.02)
Employment		
Employed (ref)		-
Unemployed		−4.31 (−3.04, −5.57) ***
Stroke		
No (ref)		-
Yes		−2.63 (−1.15, −4.11) **
High Blood Pressure		
No (ref)		-
Yes		−1.51 (−0.30, −2.72) *
Emphysema		
No (ref)		-
Yes		−2.82 (−0.53, −5.12) *
Chronic Pain		
No (ref)		-
Yes		−4.50 (−3.50, −5.50) ***
Arthritis		
No (ref)		-
Yes		−3.77 (−2.52, −5.02) ***
Asthma		
No (ref)		-
Yes		−0.98 (−2.43, 0.47)
High Cholesterol		
No (ref)		-
Yes		−0.99 (−2.03, 0.05)
Coronary Heart Disease		
No (ref)		-
Yes		−2.36 (−4.76, 0.05)
Angina		
No (ref)		-
Yes		−0.35 (−2.41, 1.71)
Heart Attack		
No (ref)		-
Yes		2.58 (0.48, 4.68) *
Heart Disease		
No (ref)		-
Yes		−1.99 (−4.67, 0.69)
Other Heart Disease		
No (ref)		-
Yes		−1.39 (−0.09, −2.70) *
Bronchitis		
No (ref)		-
Yes		−0.77 (−3.00, 1.47)
Cancer		
No (ref)		-
Yes		−2.59 (−1.17, −4.00) ***

* *p* < 0.05, ** *p* < 0.01, *** *p* < 0.00.

**Table 4:** Unadjusted and adjusted relationship between food insecurity and mental component of quality of life.

	Unadjusted	Adjusted
	Estimate (95% CI)	Estimate (95% CI)
Food Security Status		
Food Secure (ref)	-	-
Food Insecure	−8.75 (−7.45, −10.06) ***	−5.37 (−4.06, −6.68) ***
Age		
18–44 (ref)		-
45–64		1.96 (0.54, 3.39) **
65+		5.67 (4.01, 7.33) ***
Gender		
Male (ref)		-
Female		−0.85 (−0.08, −1.62) *
Education		
<High School (ref)		-
High School Diploma/GED		1.36 (0.22, 2.50) *
College		1.33 (0.17, 2.49) *
Race/Ethnicity		
Non-Hispanic White (ref)		-
Non-Hispanic Black		1.30 (0.28, 2.31) *
Hispanic		0.10 (−1.09, 1.29)
Other		−0.78 (−2.26, 0.71)
Marital Status		
Married (ref)		-
Widow/Divorce/Separated		−1.15 (−0.28, −2.02) *
Never Married		0.76 (−0.36, 1.88)
Region		
Northeast (ref)		-
West		1.10 (−0.39, 2.58)
South		0.17 (−1.23, 1.57)
Midwest		−0.05 (−1.50, 1.40)
Poverty		
Poor/Near Negative (ref)		-
Near Poor		0.19 (−1.66, 2.04)
Low Income		1.77 (0.20, 3.34) *
Middle Income		1.87 (0.36, 3.38) *
High Income		2.70 (1.01, 4.40) **
Insurance		
Private (ref)		-
Public		−1.76 (−0.68, −2.84) **
Uninsured		−0.29 (−2.70, 2.12)
Employment		
Employed (ref)		-
Unemployed		−2.54 (−1.50, −3.58) ***
Stroke		
No (ref)		-
Yes		−0.46 (−1.76, 0.83)
High Blood Pressure		
No (ref)		-
Yes		−0.77 (−1.79, 0.25)
Emphysema		
No (ref)		-
Yes		−1.23 (−3.09, 0.63)
Chronic Pain		
No (ref)		-
Yes		−2.37 (−1.50, −3.24) ***
Arthritis		
No (ref)		-
Yes		−1.31 (−0.33, −2.28) **
Asthma		
No (ref)		-
Yes		−1.06 (−2.28, 0.17)
High Cholesterol		
No (ref)		-
Yes		0.36 (−0.62, 1.34)
Coronary Heart Disease		
No (ref)		-
Yes		1.99 (−0.49, 4.46)
Angina		
No (ref)		-
Yes		−0.71 (−2.83, 1.42)
Heart Attack		
No (ref)		-
Yes		1.59 (−0.27, 3.45)
Heart Disease		
No (ref)		-
Yes		−3.84 (−6.65, −1.03)
Other Heart Disease		
No (ref)		-
Yes		0.59 (−0.55, 1.72)
Bronchitis		
No (ref)		-
Yes		−0.10 (−2.52, 2.31)
Cancer		
No (ref)		-
Yes		−0.20 (−1.20, 0.80)

* *p* < 0.05, ** *p* < 0.01, *** *p* < 0.00.

## Data Availability

The dataset generated and analyzed during the current study is publicly available and can be accessed on the Medical Expenditure Panel Survey (MEPS) website at https://meps.ahrq.gov/data_stats/download_data_files.jsp (accessed on 30 September 2024).
